# Ultrastructural examination of cryodamage in *Paracentrotus lividus* eggs during cryopreservation

**DOI:** 10.1038/s41598-024-57905-2

**Published:** 2024-04-15

**Authors:** S. Campos, J. Troncoso, E. Paredes

**Affiliations:** Centro de Investigación Mariña (CIM), Departamento de Ecoloxía e Bioloxía Animal, Grupo ECOCOST, Universidade de Vigo, 36208 Pontevedra, Spain

**Keywords:** Scanning electron microscopy, Transmission electron microscopy, Biodiversity, Fisheries

## Abstract

This study examinates the challenges of cryopreserving sea urchin (*Paracentrotus lividus*) eggs, a task hindered by factors like low membrane permeability and high sensitivity to cryoprotective agents (CPAs). While successful cryopreservation has been achieved for some marine invertebrates, eggs remain problematic due to their unique characteristics. The study explores the impact of various CPAs and cryopreservation techniques on sea urchin eggs, employing scanning and transmission electron microscopy to analyze cellular damage. The findings reveal that exposure to low CPA concentrations (0.5 M) did not induce significant damage to eggs. However, high concentrations (3 M) proved highly detrimental. Every cryopreservation approach investigated in this study resulted in irreversible damage to the sea urchin eggs, rendering them nonviable for future use. The research sheds light on the importance of understanding the structural alterations induced by CPAs and cryopreservation methods. This knowledge is essential for refining cryopreservation methods, potentially paving the way for successful preservation of these challenging cells.

## Introduction

Cryopreservation, a process involving the freezing, banking, and thawing of living organisms, cells, or tissues with cryoprotecting agents (CPAs)^1^, has been widely utilized in animal breeding, conservation, and more recently in aquaculture and genetic programs. While extensively studied in land animals^2–4^, recent decades have seen significant progress in the cryopreservation of aquatic species, both marine and freshwater^5–7^. Marine invertebrates, such as mussels and oysters, can be cryopreserved^8–10^, but the majority of developed protocols focus on sperm or larvae due to the challenges associated with preserving eggs. The complexities arise from the unique characteristics of aquatic organism eggs, including their large volume, low surface/area ratio, high water and lipid content, low permeability to water and CPAs, sensitivity to CPA toxicity, and susceptibility to chemicals and chilling^6^. Currently, cryopreservation efforts for marine invertebrates are limited to a small number of species that hold economic significance in aquaculture production^11,12^ and has been focused on late-stage embryos and larvae^13,14^.

The sea urchin *Paracentrotus lividus,* our specie of study, is a model organism for research in developmental biology. It is a species with high economic value and interest in their management and aquaculture has increased greatly in recent years. It also has high ecological importance, as it plays a major role in functioning, dynamics and structure of benthic assemblages. Its distribution goes throughout the Mediterranean Sea and European Atlantic coast^15^. In general, sea urchin eggs usually do not exceed 80–100 μm in diameter^16^ and in the case of *P. lividus* the average egg diameter is 90 μm.

Sea urchin eggs haven’t been successfully cryopreserved so far due to several factors such as cell membrane composition and its low permeability, which makes movement of CPA and water slower. Another notable characteristic is that sea urchins exhibit osmoconformity, wherein alterations in the osmolarity of their bodily fluids correspond directly to fluctuations in the salinity of the surrounding environment. Additionally, sea urchins display high sensitivity to chemicals, and the introduction of cryoprotective agents (CPAs) might potentially elicit toxic responses. Paredes and Bellas^17^ conducted a research investigation on the sea urchin species *Paracentrotus lividus*, evaluating various cryoprotective agents (CPAs) and their potential toxicity. Results indicated that the unfertilized egg exhibited the highest sensitivity among developmental stages, while the blastula demonstrated the greatest tolerance to the tested CPAs^16^.

Unfertilized sea urchin eggs are surrounded by a gelatinous envelope of glycoprotein called the jelly coat followed by the vitelline layer, under which is a plasma membrane. In mature eggs the plasma membrane is covered with many papillae or microvilli^18^. Microvilli are important in fertilization, as they may be sites for initial contacts between female and male gametes^19,20^. Directly under the plasma membrane a layer of cortical secretory vesicles or cortical granules is located, randomly distributed and tightly adhered to the plasma membrane. Cortical granules are membrane bound vesicles formed by the Golgi apparatus of the egg that migrate to the cell periphery during the later stages of oogenesis. At fertilization there are 2 main sperm-induced Ca^2+^ events, first a cortical calcium release (Cortical Flash)^21,22^, secondly a wave of cytoplasmic calcium travel through the egg from the point of sperm penetration, propagates over the entire egg and triggers a wave of exocytosis of the cortical granules causing their membranes to fuse with the plasma membrane of the egg. Upon exocytosis, the enzymes and structural proteins contained in the cortical granules modify the egg vitelline layer and produce a chemically and mechanically stable membrane: the fertilization membrane, which prevents polyspermy^23–28^.

The aim of this study is to examine the damage in *Paracentrotus lividus* eggs caused by different cryoprotecting agents and cryopreservation methods, using electron microscopy techniques (scanning (SEM) and transmission electron microscopy (TEM)). By analyzing the obtained images, we expect to better understand the impact of current protocols on the eggs and determine the damage produced. Understanding cryodamage will help us design better cryopreservation protocols for these recalcitrant cells.

## Results

### Characterization of control eggs

**Figure 1:**
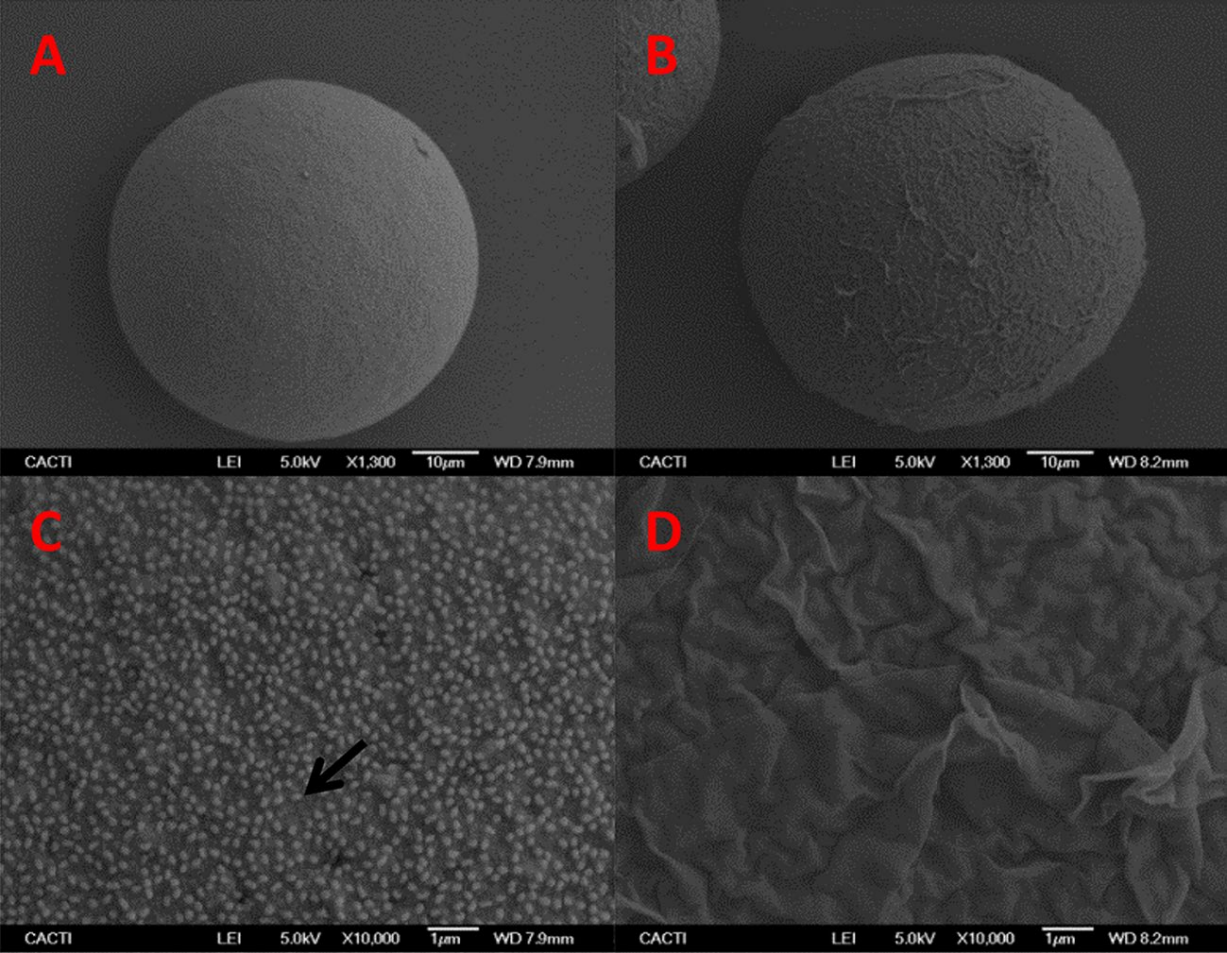
SEM images of unfertilized (**A**) and fertilized (**B**) control eggs. Detail of microvilli (black arrow) and fertilization membrane showing in (**C**) and (**D**), respectively.

SEM images revealed that unfertilized control eggs exhibited perfectly round shape with diameters of 80–100 μm and they were covered by numerous microvilli (≈3,70,000) (Fig. 1A,C). The fixation procedure removes the jelly coat surrounding sea urchin eggs, so microvilli are visible whereas in living conditions would be covered by the gelatinous layer. Conversely, fertilized eggs also present a spherical shape, but they possess a fertilization membrane covering the egg (Fig. 1B,D).

**Figure 2:**
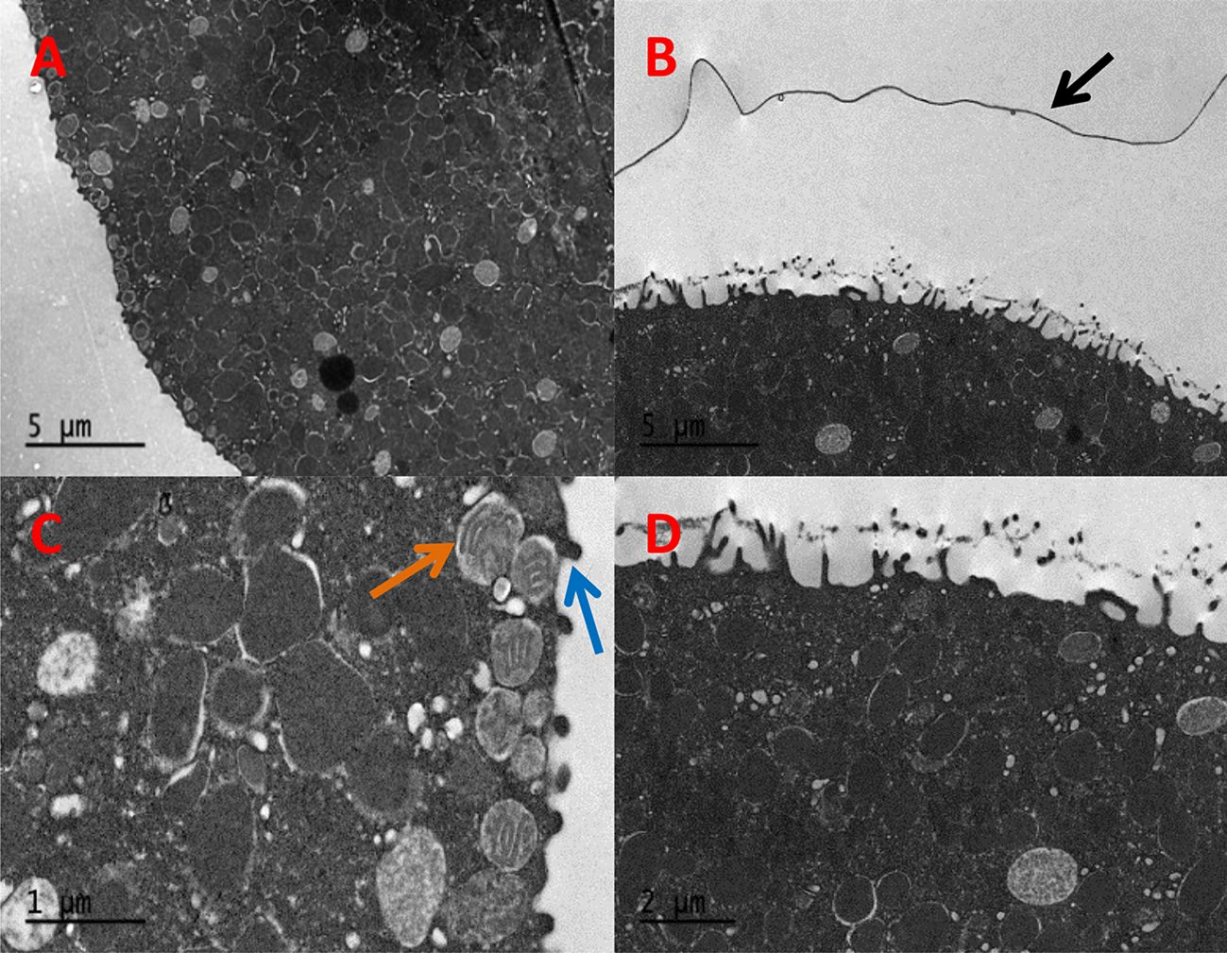
TEM images of unfertilized (**A**) and fertilized (**B**) control eggs. Fertilization membrane lifted (**B**, black arrow) detail of microvilli (**C**, blue arrow) cortical granules (**C**, orange arrow), and elongated microvilli (**D**).

In TEM images, unfertilized eggs display round vesicles located right under de plasma membrane, known as cortical granules (Fig. 2A,C). These vesicles are involved in the fertilization process and the lifting of the fertilization membrane^29–33^. After fertilization, cortical granules disappear, the fertilization membrane is visible, and the microvilli have elongated (Fig. 2B,D). In both cases the cytoplasm is compact, well organized and homogeneous cellular material and organelles, such as clusters of mitochondria (Supplementary data Fig. 9) and yolk platelets.

### Toxicity tests

**Figure 3:**
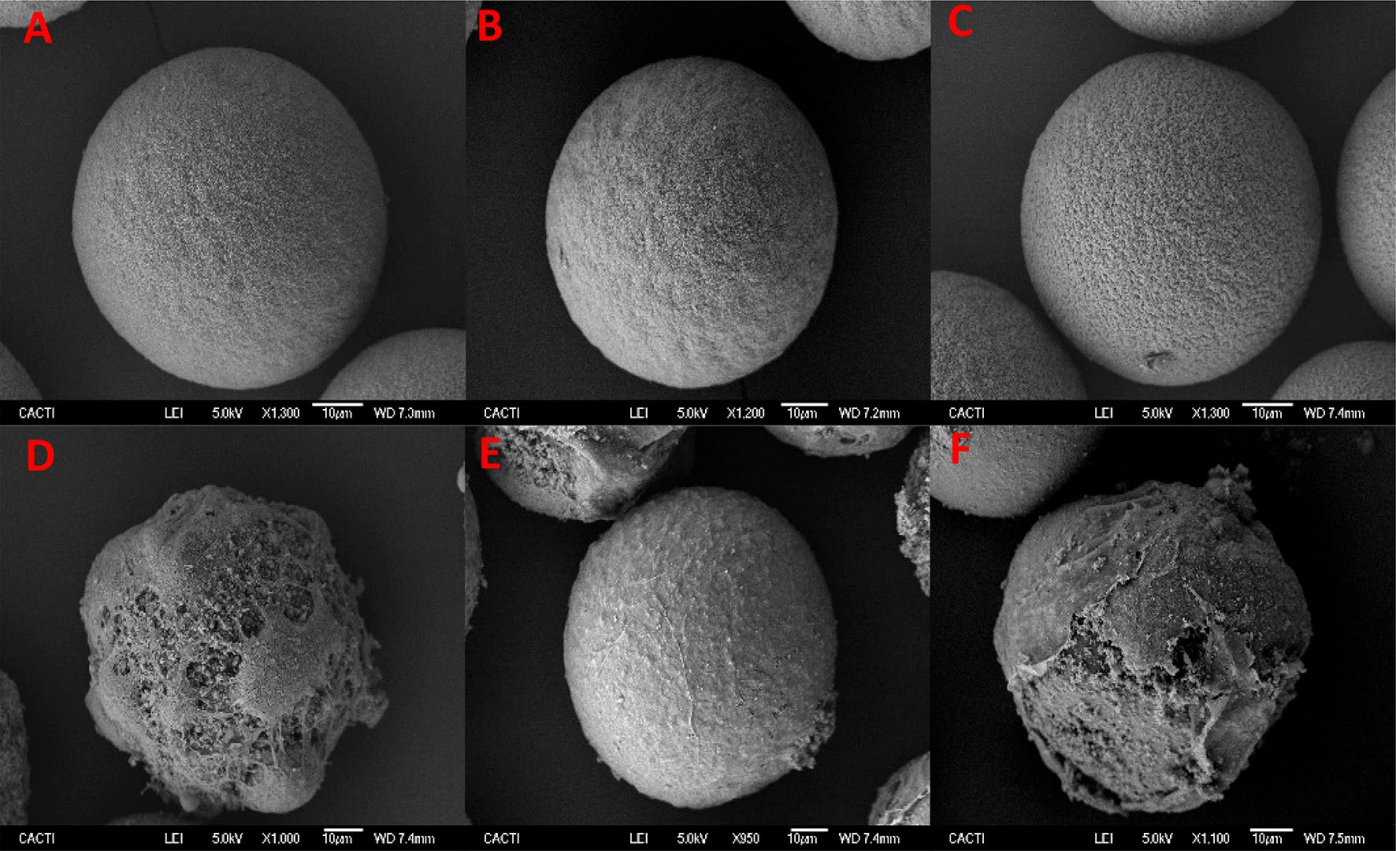
SEM images of eggs incubated for 15 min adding 0.5 M DMSO (**A**), EG (**B**), PG (**C**), 3 M DMSO (**D**), EG (**E**) and PG (**F**) in 15 steps.

**Figure 4:**
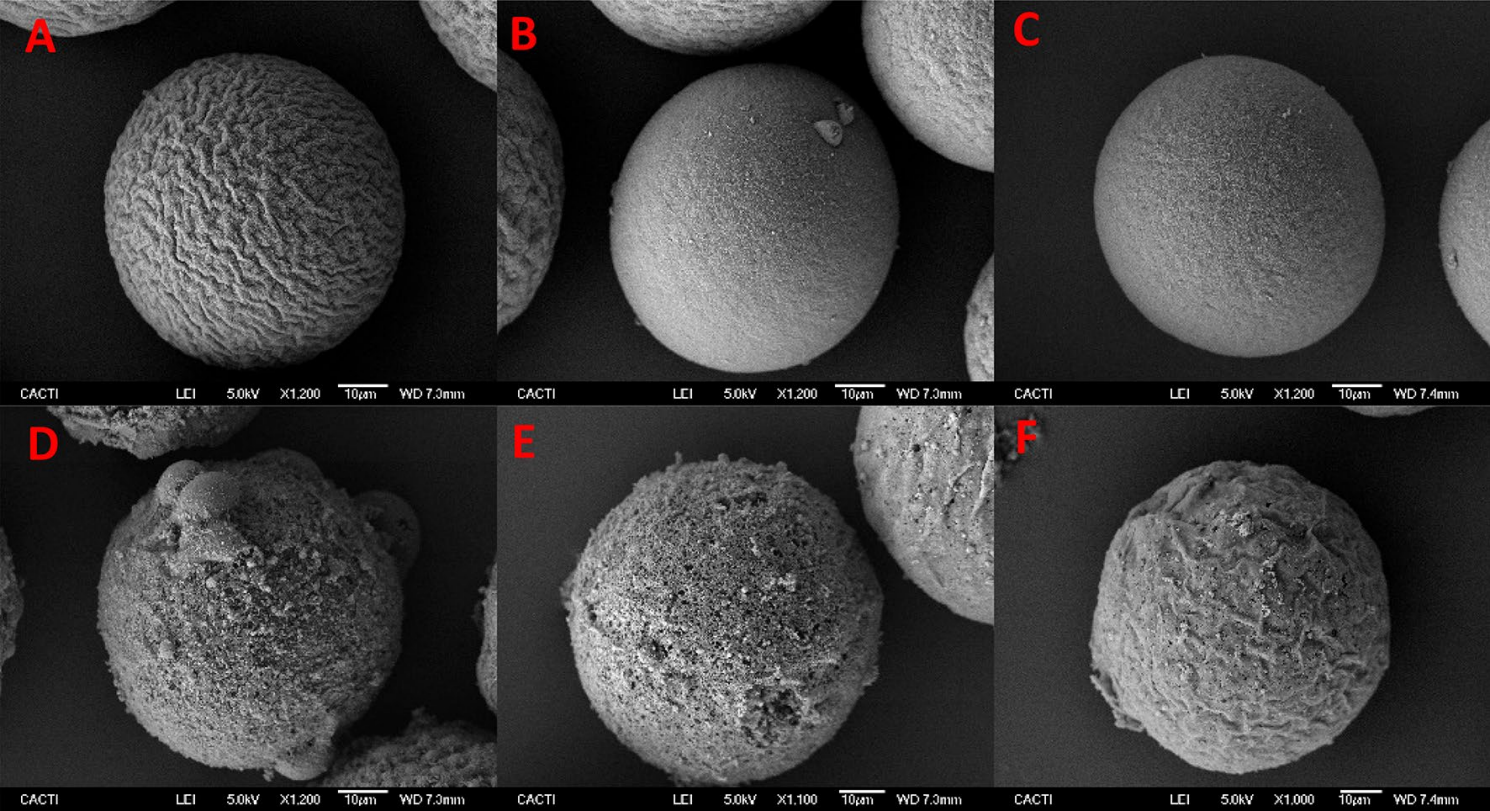
SEM images of eggs incubated for 15 min adding 0.5 M DMSO (**A**), EG (**B**), PG (**C**), 3 M DMSO (**D**), EG (**E**) and PG (**F**) in 1 step.

The SEM images revealed that when eggs were exposed to low concentrations of CPAs such as, 0.5 M Dimethyl Sulfoxide (DMSO), Ethylene Glycol (EG) and Propylene Glycol (PG) for 15 min (On-step addition) or adding the CPA in 15 equimolar steps of 1 min, minimal damage occurred to the cells. Eggs maintain their spherical shape, the microvilli are visible on the surface similarly to control cells (Figs. 3A–C, 4A–C). On the other hand, exposing the eggs to CPA concentrations such as 3 M, regardless of the addition method used, caused an irreparable damage independently the CPA nature. (Figs. 3D–F, 4D–F). High concentrations of CPAs caused significant harm rendering the cells unable to recover or maintain their integrity.

**Figure 5:**
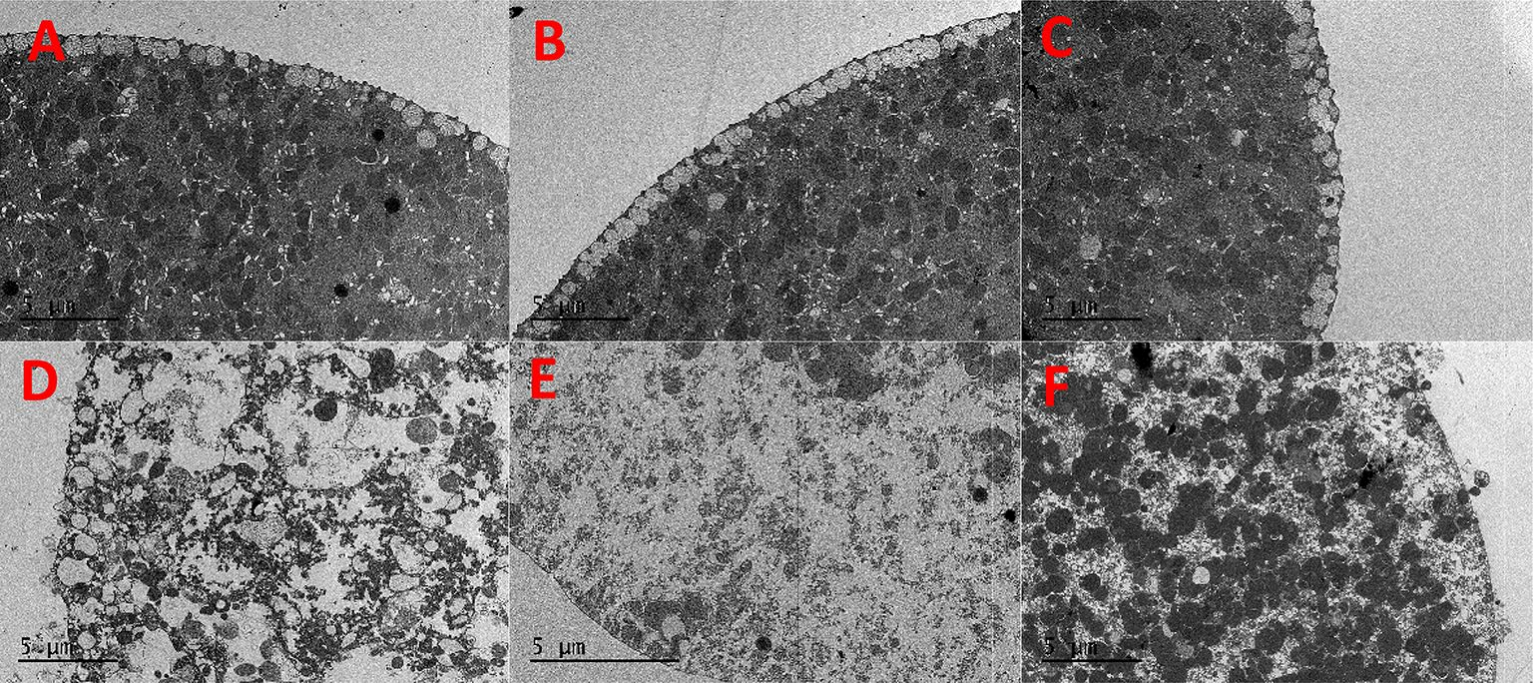
TEM images of eggs incubated for 15 min adding 0.5 M DMSO (**A**), EG (**B**), PG (**C**), 3 M DMSO (**D**), EG (**E**) and PG (**F**) in 15 steps.

**Figure 6:**
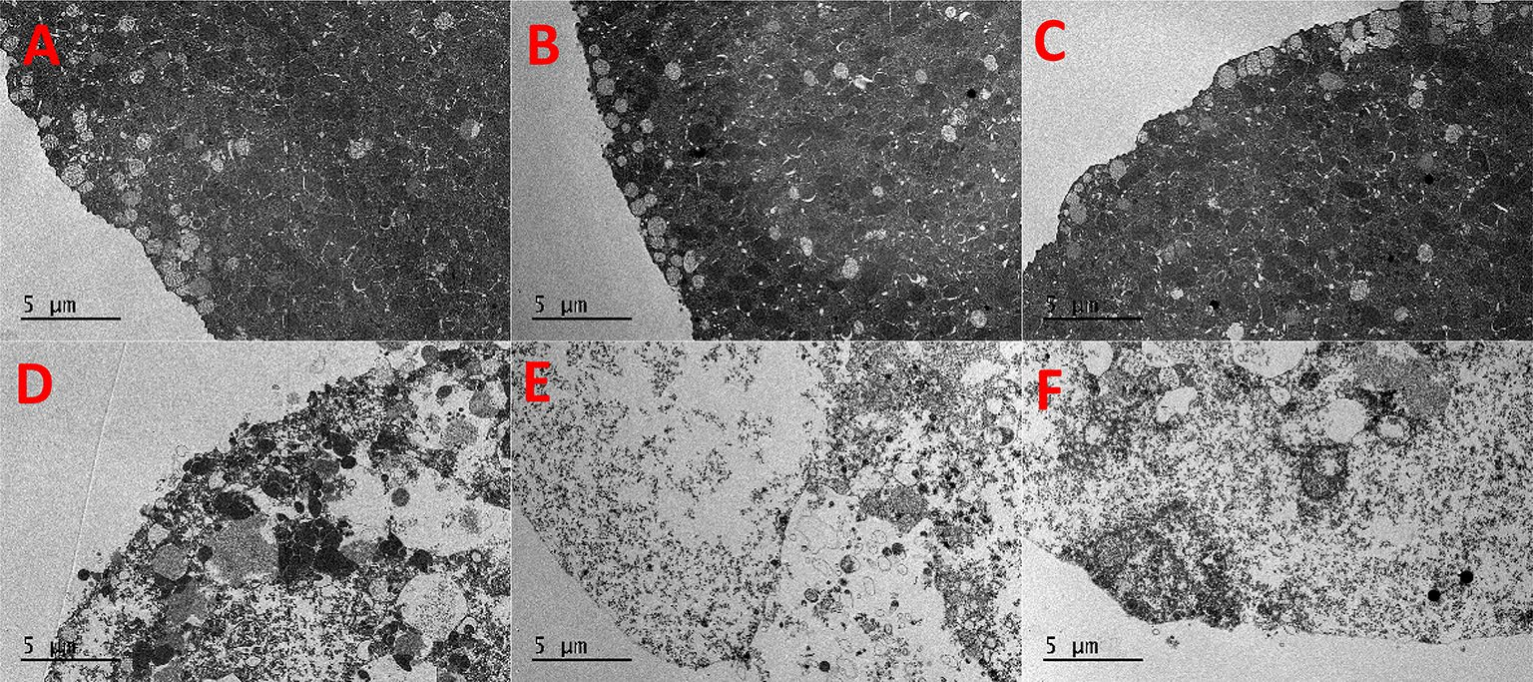
TEM images of eggs incubated for 15 min adding 0.5 M DMSO (**A**), EG (**B**), PG (**C**), 3 M DMSO (**D**), EG (**E**) and PG (**F**) in 1 step.

In TEM images, the cells exposed to low concentrations of CPAs still presented visible microvilli and cortical granules similar to the controls (Figs. 5A–C, 6A–C). On the other hand, cells exposed to 3 M CPAs showed evident signs of damage, with the intracellular material appearing disorganized (Figs. 5D–F, 6D–F). The damage is substantial internally with a disrupted internal organization and cell functioning that matches the external damage observed with SEM.

When comparing the structural integrity of the cells exposed to CPAs, even at the low concentration (in this case 0.5 M) it appears that the cells, both externally and internally, maintain their integrity better with structures well defined, cohesive and well organized when the CPA exposure is carried out stepwise rather than in one single step (in both cases the total duration of the exposure was 15 min at room temperature).

### Cryopreservation methods

#### Slow cooling

**Figure 7:**
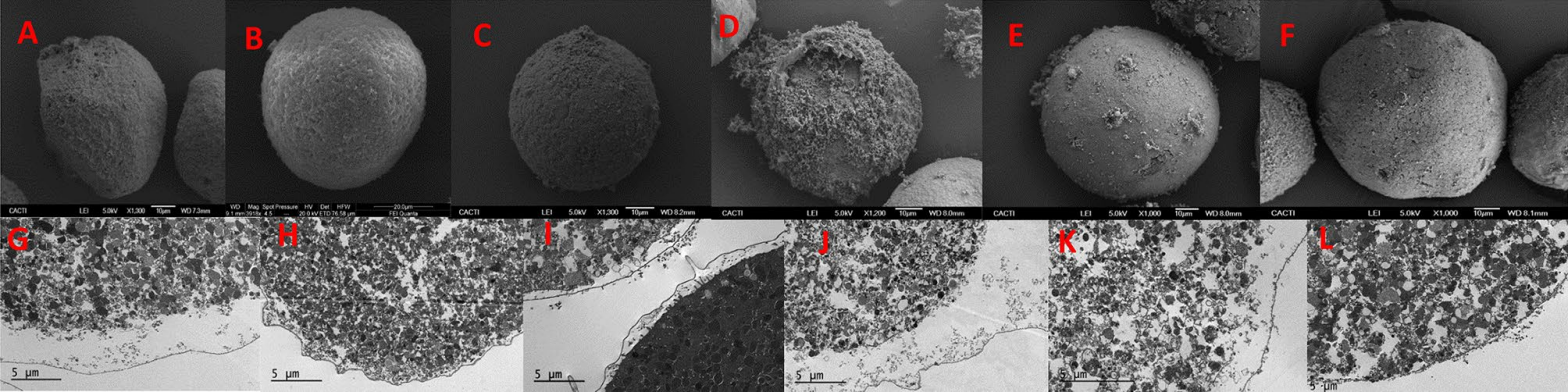
SEM images of eggs cryopreserved using slow cooling with DMSO 0.5 M (**A**); 1 M (**B**); 1.5 M (**C**); 2 M (**D**); 2.5 M (**E**) and 3 M (**F**). TEM images of unfertilized eggs cryopreserved using slow cooling with DMSO 0.5 M (**G**); 1 M (**H**); 1.5 M (**I**); 2M (**J**); 2.5 M (**K**) and 3 M (**L**).

Scanning Electron Microscopy images have shown that the cryopreservation method employed for slow cooling resulted in evident damage to the eggs, regardless of the DMSO concentration used (Fig. 7). Furthermore, the combination of DMSO with non-permeant CPAs did not yield improvement (Supplementary data Fig. 1). The SEM images display ruptured membranes and the extrusion of cellular material. Slow cooling led to irreparable damage to the eggs.

TEM images have revealed important intracellular damage, including disrupted membranes, organelles. The absence of cortical granules around the cell. The presence of the fertilization membrane even though there was no fertilization has taken place is related to the vesicles rupture during cooling (Fig. 7). Cryopreservation induced damage has provoked the cell to send the wrong signals though a Ca^2+^ pulse as if fertilization had taken place and thus the lifting of the fertilization envelope. Remarkably, regardless of the concentration of DMSO used or its combination with non-permeant CPAs, the outcome consistently manifested as highly damaged cells beyond any possibility of repair (Supplementary data Fig. 2).

The addition of non-permeating CPAs like Trehalose (TRE), Polyvinylpyrrolidone (PVP), Sucrose (SUC) or Bovine Serum Albumin (BSA) alongside DMSO did not produce any significant improvement in the outcome of the slow cooling as it can be seen in (Supplementary Data Fig. 2).

#### Vitrification

**Figure 8:**
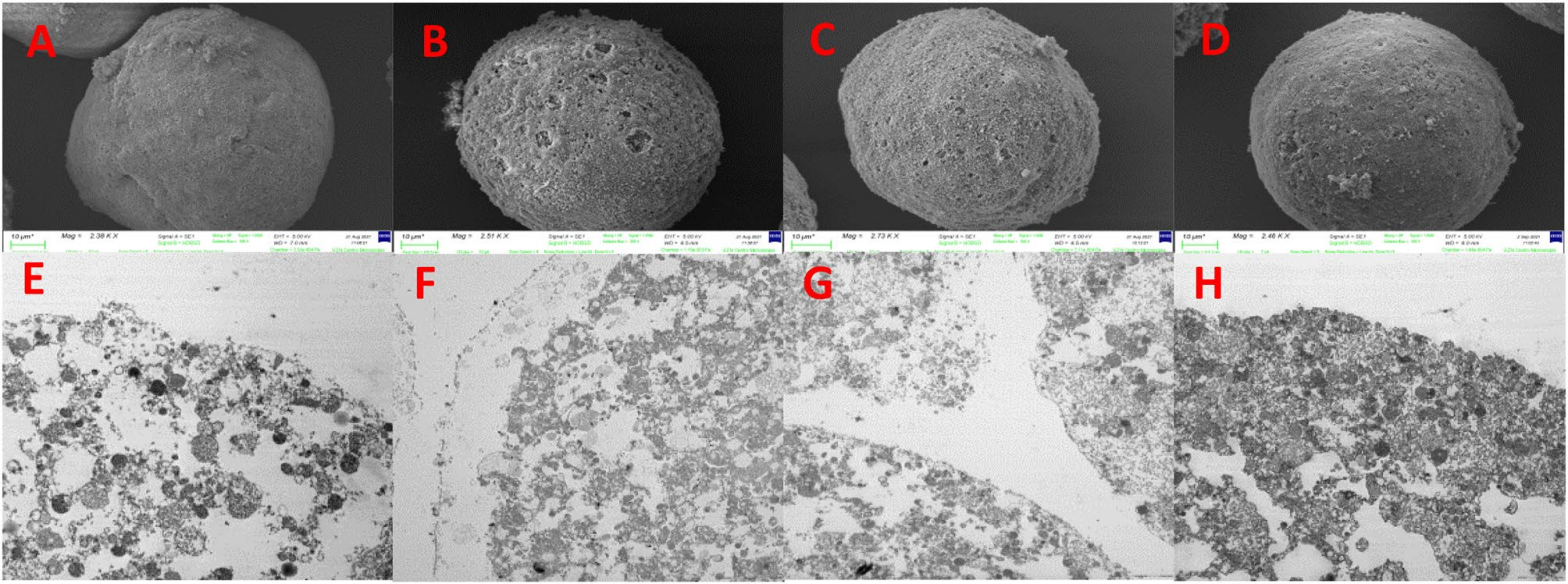
SEM images of eggs cryopreserved using vitrification by contact with DMSO 0.5 M (**A**) and 3 M (**B**) or EG 0.5 M (**C**) and 3 M (**D**). TEM images of eggs cryopreserved using vitrification by contact with DMSO 0.5 M (**E**) and 3 M (**F**) or EG 0.5 M (**G**) and 3 M (**H**).

Independently of the three methods of vitrification used the results have been the same. The application of Vitrification either using DMSO or EG in varied concentrations up to 3 M did not prove to be effective for the cryopreservation of *P. lividus* eggs, resulting in severe damage. A fibrous layer was observed covering the eggs likely formed due to the exocytosis of material and vesicles. The egg integrity is compromised as the membranes are damaged (broken), and exocytosed vesicles and material are on the surface as seen in Fig. 8 (see supplementary data Figs. 3, 5, 7 for all concentrations and vitrification methods).

When using EG (1.5 M), the ultrastructure of the cytoplasm appeared to be in relatively better conditions (Supplementary Data Fig. 4F) but the surface of the cell remained adversely affected. Again, with the other concentrations of cryoprotectants, the internal structure of the cell appeared completely dismantled and the fertilization membrane is present even though the eggs used for cryopreservation were not fertilized (Supplementary Data Figs. 4, 6, 8).

## Discussion

Cryopreservation of aquatic organisms is relatively recent and its combination with ultrastructural observations are even more recent^34^. Successful cryopreservation is dependent on several factors such as membrane permeability^35,36^, CPA toxicity and capacity of cryoprotection^37^, addition and removal of CPAs^36^, cryoinjury^38^ intracellular ice formation (IIF), chilling injury or cooling and warming rates^12^.

The aim of this study was to obtain knowledge about the damage caused by exposing the eggs to different concentrations of Dimethyl Sulfoxide, Ethylene Glycol and Propylene Glycol for 15 min, and to evaluate de damage caused by four different cryopreservation techniques, both using slow cooling and vitrification.

In general, with exposure to CPAs and cryopreservation, widespread damage to the cell surface structure has been observed, with extrusion of vesicles, disappearance of microvilli, and probably denaturation of actin filaments. This damage is also found in the cytoplasm with the disappearance of cortical granules, which causes fertilization membrane lifting. Crucially, the process of egg activation can occur without sperm. Eggs can be activated by various nonspecific treatments that trigger the same or similar intracellular events that would normally be initiated by sperm entry^39^.

Results have shown that exposing the eggs to low concentrations (0.5 M) of DMSO, EG and PG did not cause damage to the cells (Figs. 3, 4, 5, 6A–C) which is in agreement with prior data of CPA toxicity^17,40^*.* In the case of eggs, the NOEC (No Observed Effect Concentration) values calculated support the results obtained in this study, since after exposing those cells to low concentrations of CPA, the ultrastructure was similar to controls. But this low concentration is not enough to protect the cells during cryopreservation. On the other hand, high concentrations of CPAs (3 M) caused great damage and the cells were not available even before cryopreservation (Figs. 3D–F, 4D–F, 5D–F, 6D–F). These results are consistent with the LOEC (Lowest Observed Effect Concentration) values calculated by Paredes and Bellas^17^ and Paredes et al.^40^ for DMSO, EG and PG.

SEM and TEM images have not shown many differences between different CPAs. At low concentrations (0.5 M) eggs presented minimal damage, with intact microvilli and cortical granules still visible, similar to controls. On the other hand, high concentrations (3 M) caused irreversible harm (broken membranes, internal disorganization) regardless of the inherent characteristics of the CPA. However, when comparing addition methods, stepwise addition showed cells maintaining integrity better (externally and internally) than those exposed in a single step. This is in agreement with previous research^17,41^ which indicated that osmotic shock could occur using the single step method. Therefore, at this point we know that the cytotoxicity of the CPAs is a major source of damage for the sea urchin eggs.

All cryopreservation methods used in this study have caused an irreparable damage to the sea urchin eggs, although one of them seemed to show a better outcome. At TEM using vitrification by contact (CMV kit) 1.5 M EG, the ultrastructure of the cytoplasm is better preserved (Supplementary data Fig. 4F). But SEM observations indicated that the egg surface has also been damaged. So, this is a clear example indicating that the observation of the topography of the egg surface is essential to have information on the actual conditions of the eggs. Only TEM observations are not sufficient to drive conclusions.

The egg surface is the region of the cell that reacts appropriately with the sperm if the physiological conditions of the eggs are optimum^42^. Eggs have specific organelles and vesicles (cortical granules, acidic vesicles) as well as membrane receptors, glycoproteins and actin filaments essential for fertilization^28^. For example, there is a species-specific trypsin-sensitive glycoprotein called "sperm receptors" on the membrane, responsible for the recognition and attachment of the BINDIN (protein located in sperm acrosomal vesicle responsible for fusion of the sperm and egg plasma membranes)^26,43^. Actin filaments are present within the microvilli and they are also observed along the inner surface of the plasma membrane, frequently situated between adjacent microvilli, also needed to acrosomal processes^19,44–49^. So, with all these cryopreservation methods we have destroyed the membrane structure therefore the cell will no longer be viable.

Successful vitrification depends, among other factors^20^, on whether the CPAs solution vitrifies. According to our data, none of the CPAs concentrations and combinations used in this study achieved vitrification. The problem associated with these results is that if higher concentrations of cryoprotectants are needed to obtain survival, this will damage the cells through chemical toxicity and osmotic shock The extent to which the solution vitrifies or turns into a glassy state depends on two main factors: the concentration of solutes and the cooling rate^50^. In this work we haven′t found the right balance between rapid cooling and warming and low toxicity of vitrification solutions, nonetheless by examining the cryodamage produced we can gain insights for new protocol development.

Slow cooling is not going to cryopreserve sea urchin eggs^35,51–54^, Vitrification seems to be the way. Vitrification usually requires high concentration of CPAs (producing toxicity and osmotic shock) or high dehydration, which sea urchin eggs do not tolerate (unpublished data). It is necessary to find the right balance between CPA concentration and cryopreservation outcome. In the context of cryopreservation or vitrification, controlling the water content of the cells is crucial. If the water content is not properly managed, ice crystal formation can occur during freezing, leading to cell damage, so cell dehydration is also an important factor to be considered^1,55^. Sea urchin eggs have a large amount of intracellular water (30%^56^). When water is removed from cells, osmotic stress can occur. However, this stress can be managed and controlled during the dehydration process. It′s important to find the right balance between dehydration and osmotic stress to avoid damaging the cells. Olver et al.^56^ have shown that an osmotic damage model with a time-dependent aspect implies that both the nature of the solution used and the duration of exposure to osmotic stress play significant roles in ensuring osmotic survival. For example, toxicity tests using DMSO up to 1.4 M added in 15 equimolar steps have shown normal development to pluteus larvae after 48 h incubation. Higher concentrations have resulted in no egg survival as no development was found (unpublished data).

Another critical aspect of cryopreservation is the addition/removal of CPAs. Their addition is important for preserving biological materials during freezing, while their proper removal is crucial for maintaining cell viability and functionality when these materials are used or thawed. The choice will depend on the specific application, the type of biological material being preserved, and the goals of the preservation process. It seems that this procedure needs to be stepwise as single-step addition or removal will result in excessive osmotic excursions and cell death^17,41,53^, but we may have to design better methods by modeling.

This is the first time that structural damage in *P. lividus* eggs caused by different CPAs and after cryopreservation and vitrification methods has been studied. Ultrastructural and cellular surface observations can improve the understanding of the mechanisms involving cryopreservation processes and cryodamage. Future research will involve the modelling and detailed study of cocktails of CPAs and even faster cooling and warming rates like in the case of Vitrification and ultrafast laser working that might allow us to achieve survival with lower CPA concentration as reported by Seki et al., Jin and Mazur and Jin et al.^57–59^.

## Materials and methods

### Collection of biological material

Eggs and sperm were collected from sea urchins provided by ECIMAT-Marine station marine culture service, sampled from Ria de Vigo (NW-Spain). Gamete quality was assessed by observing spherical shape and brownish homogeneous color of the cytoplasm for the eggs and motility for sperm under the microscope. Control eggs were fertilized and no females with less than 90% fertilization rate were used.

### Toxicity of the cryoprotecting agents (CPAs)

Eggs were exposed to concentrations of 0.5 M and 3 M DMSO, EG or PG prepared at double the final concentration. Toxicity tests were done by mixing 1 mL of eggs with 1 mL of CPA (1:1 dilution) either in one single step and incubate for 15 min or adding the CPA in 15 equimolar steps of 1 min^17^. In the one step method after those 15 min samples were washed out with Filtered Sea Water (FSW, 0.22 µm + UVA). In the 15 steps method after equilibration the CPA was diluted stepwise with FSW in 12 steps 1 min apart following guidelines by Paredes and Bellas^17^. Finally, samples were fixed for electron microscopy.

### Cryopreservation methods

Different freezing methods and CPAs concentrations were used to cryopreserve unfertilized and fertilized eggs (Table 1). Samples were prepared by mixing 1 mL of eggs with 1 mL of CPA (as reported in “Toxicity tests”) in one single step and allowing an equilibration time of 5 min.

- Slow cooling, using a controlled rate freezer (Cryologic LLD, Australia). Cooling was programmed to start at 4 °C for 2 min and cooling at 1 °C/min until – 35 °C with a seeding point at – 12 °C. Then the vials were plunged to LN_2_ and thawing was done using a water bath at 20 °C (protocol modified from Bellas and Paredes^11^) The CPA concentrations used were 0.5 M, 1 M, 1.5 M, 2 M, 2.5 M and 3 M DMSO, 1.5 M DMSO combined with 0.04 M Trehalose (TRE), 0.75 M Polyvinylpyrrolidone (PVP), 0.2 M Sucrose (SUC) or 1% Bovine Serum Albumin (BSA), prepared double the concentration to obtain a 1:1 final solution (as explained in “Toxicity tests”).
- Vitrification by contact, placing a drop of the samples in a Fibreplug™ and then vitrified them by touching a CVM kit™ (Cryologic LLD, Australia) submerged in liquid nitrogen. Samples were vitrified without CPAs and using 0.5 M, 1.5 M and 3 M DMSO and EG prepared as in “Toxicity tests”. Thawing was done by placing the vitrified samples into seawater at 20 °C.
- Droplet vitrification following the method published by de Vries et al.^60^. Cells were loaded in a 5 mL syringe and a second 5 mL syringe was loaded either with seawater or different CPAs concentration (0.5 M, 1.5 M and 3 M DMSO and EG, prepared double the concentration in sea water). Both syringes were connected with a tube that ended on a mixing mechanisms which had a needle in its end. This mechanism was attached in a vertical position with the needle facing down toward a box with a plastic tube and a funnel containing liquid nitrogen. Thawing was done by placing the vitrified samples into seawater at 20 °C.
- Vitrification in straws, cryopreservation was achieved by plunging a straw directly into liquid nitrogen (0.25 µL). Samples were vitrified in sea water and using 0.5 M, 1.5 M and 3 M DMSO and EG prepared double the concentration in sea water. A water bath at 20 °C was used to thaw the straws.

**Table 1:** Methods and CPA concentrations used to cryopreserve *P. lividus* eggs and obtain SEM and TEM images.

Cryopreservation method	CPA concentration	Cooling rates	Thawing rates
Slow cooling	0.5 M, 1 M, 1.5 M, 2 M, 2.5 M, 3 M DMSO 1.5 M DMSO + 0.04 M TRE1.5 M DMSO + 0.75 M PVP 1.5 M DMSO + 0.2 M SUC 1.5 M DMSO + 1% BSA	1 °C/min^11^	280 °C/min water bath at 25 °C^61^
Vitrification by contact	NONE 0.5 M, 1.5 M, 3 M MeSo_2_ 0.5 M, 1.5 M, 3 M EG	CVM™ kit 10,000 °C/min^62^	N/A
Droplet vitrification	NONE 0.5 M, 1.5 M, 3 M DMSO 0.5 M, 1.5 M, 3 M EG	3 and 5 mm droplets, 1320 and 960 °C/min respectively^60^	200 °C/min water bath at 37–40 °C^63^^a^
Straw directly into LN_2_	NONE 0.5 M, 1.5 M, 3 M DMSO 0.5 M, 1.5 M, 3 M EG	1827 °C/min^50^	2950 °C/min water bath at 25 °C^64^

^a^The thawing rates depend on the droplet size, and as these are not 100% reproducible, this value would be approximate.

### Sample fixation

All samples were fixed with a solution of 1% glutaraldehyde in sea water and send to CACTI in the University of Vigo and Stazione Zoologica Anton Dohrn in Naples (SZN) to be analyzed using Scanning Electron Microscopy (SEM) and Transmission Electron Microscopy (TEM) services.

### Sample preparation for electron microscopy

After fixation with glutaraldehyde, samples were rinsed in seawater several times to remove glutaraldehyde and a post fixation treatment was made with 1% osmium tetroxide in seawater. Then they were rinsed several times in seawater and finally in distilled water before dehydrating the samples in increasing concentrations of ethanol (starting from 30 to 100%, 10 min at 4 °C) then to propylene oxide (room temperature) and resin embedding.

After polymerization of the resin at 60 °C, semi-thin and thin sections were made with an ultramicrotome for scanning and transmission electron microscope observations.

### Animal care statement and experimental design

All methods were carried out in accordance with relevant guidelines and regulations of animal handling and care at the Universidade de Vigo. Animals handled in this study are out of scope of the regulations ECC/566/2015, nonetheless animal handling was done to the best standard and following guidelines from Paredes and Costas^65^ and Paredes et al.^40^. ARRIVE guidelines in experimental design to ensure reliability of research were followed.

### Supplementary Information

Supplementary Figures.

## Data Availability

The datasets used and/or analysed during the current study available from the corresponding author on reasonable request.
