# Supplementary material for: Ultrastructural examination of cryodamage in Paracentrotus lividus eggs during cryopreservation
Source: Sci Rep. 2024 Apr 15;14:8691. doi: 10.1038/s41598-024-57905-2 (PMC11018813; doi:10.1038/s41598-024-57905-2)
Supplement: Supplementary file 1 — Supplementary Figures. [file 41598_2024_57905_MOESM1_ESM.docx]

**SUPLEMENTARY DATA**


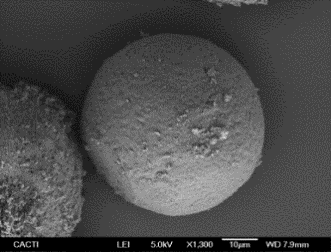

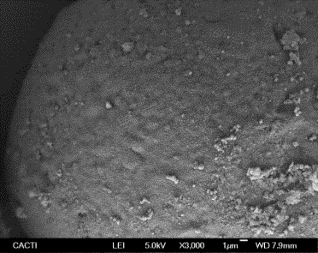


**A**

**B**


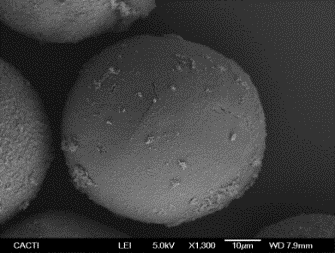

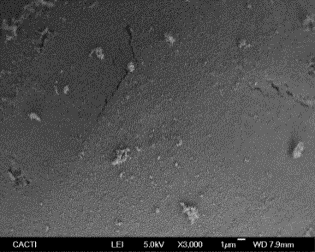


**D**

**C**


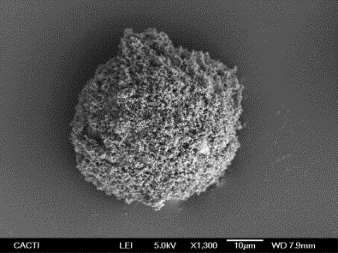

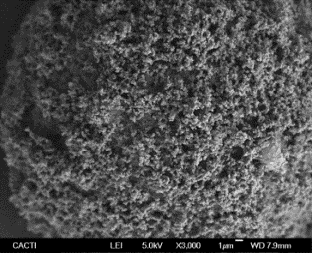


**F**

**E**


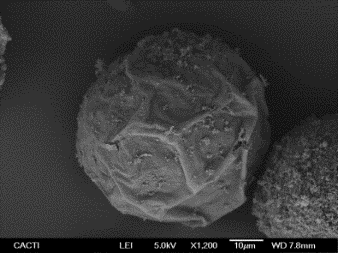

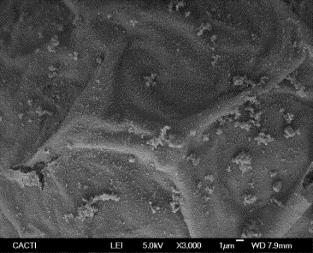


**H**

**G**

Figure 1. SEM images of eggs cryopreserved using slow cooling with DMSO 1.5M combined with TRE 0.04M (A, B: detail of A); PVP 0.75M (C, D: detail of C); SUC 0.2M (E, F: detail of E) and BSA 1% (G, H: detail of G).

**A**

**B**

**C**

**D**


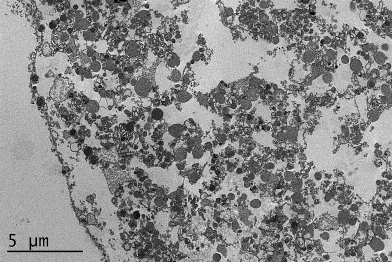

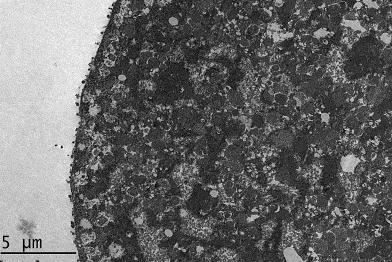

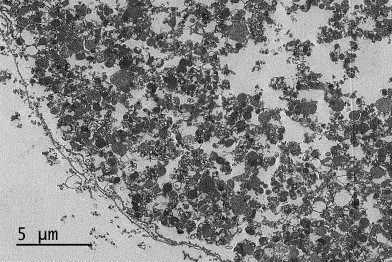

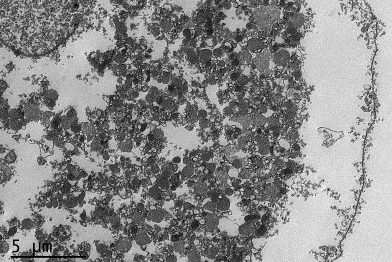


Figure 2. TEM images of unfertilized eggs cryopreserved using slow cooling with DMSO 1.5M combined with TRE 0.04M (A); PVP 0.75M (B); SUC 0.2M (C) and BSA 1% (D).


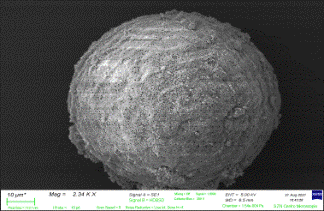


**A**


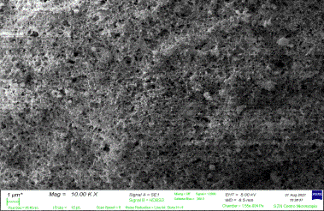


**B**


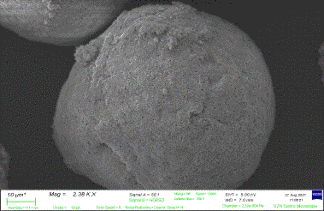


**C**


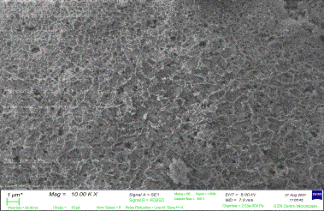


**D**


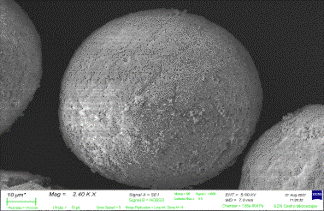


**E**


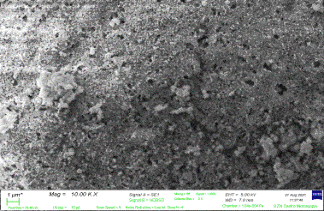


**F**


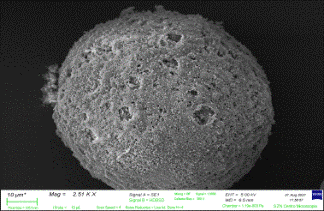


**G**


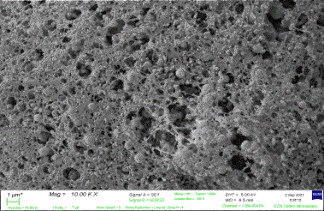


**H**


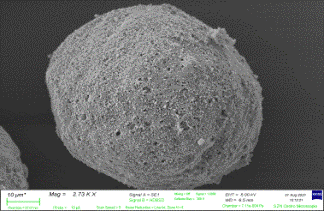


**I**


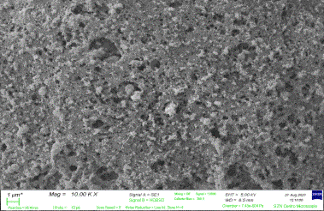


**J**


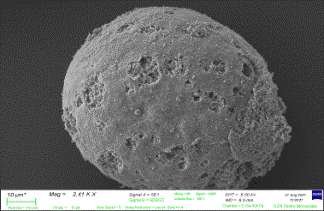


**K**


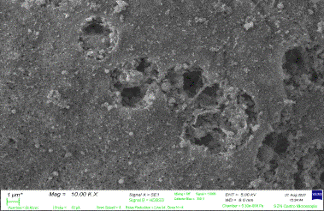


**L**


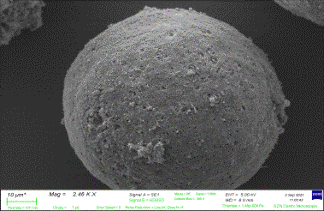


**M**


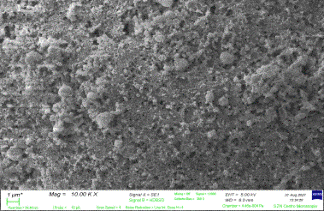


**N**

Figure 3. SEM images of eggs cryopreserved using vitrification by contact with no CPA (A, B: detail of A); DMSO 0.5M (C,D: detail of C); 1.5M (E, F: detail of E); 3M (G, H: detail of G) or EG 0.5M (I, J: detail of I); 1.5M (K,L: detail of K) and 3M (M, N: detail of M).


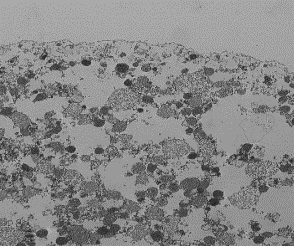

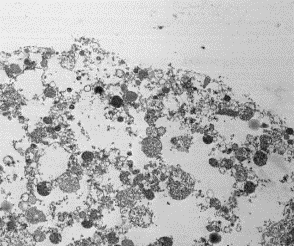

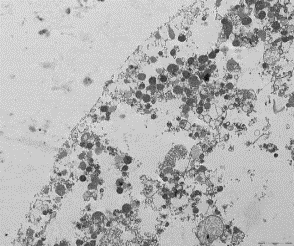

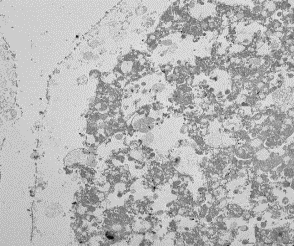

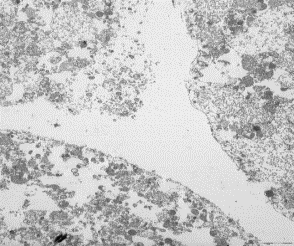

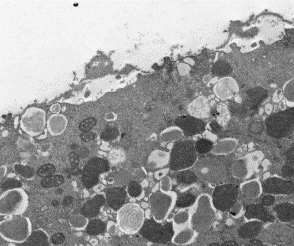

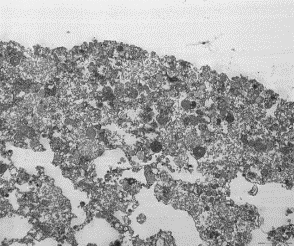


**A**

**D**

**C**

**B**

**E**

**G**

**F**

Figure 4. TEM images of unfertilized eggs cryopreserved using vitrification by contact with no CPA (A); DMSO 0.5M (B); 1.5M (C); 3M (D) or EG 0.5M (E); 1.5M (F) and 3M (G).


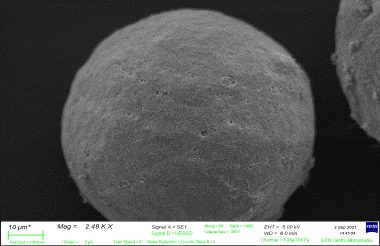

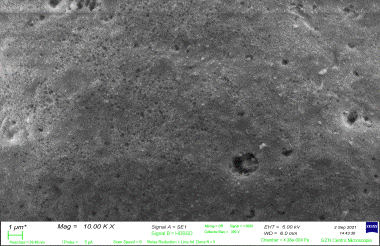

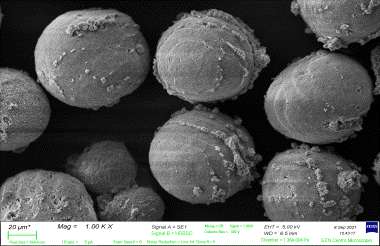

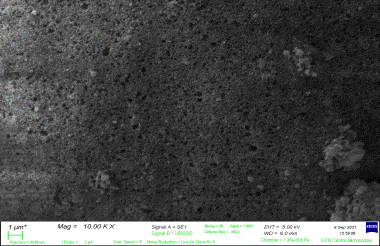

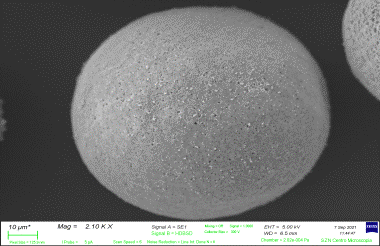

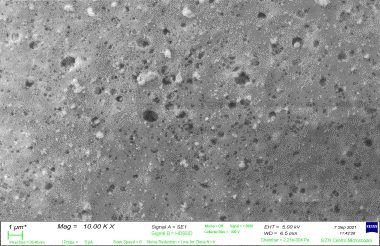

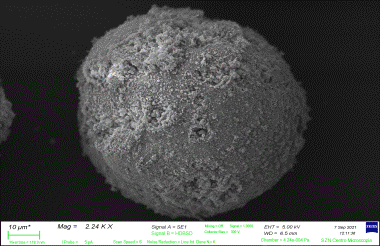

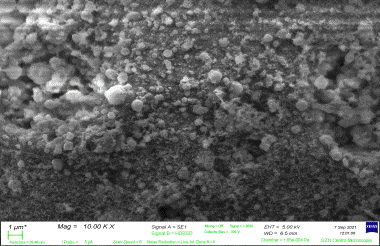

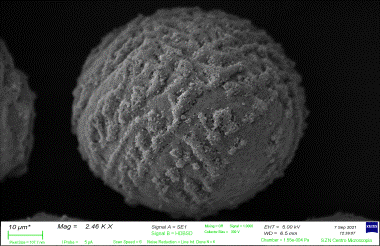

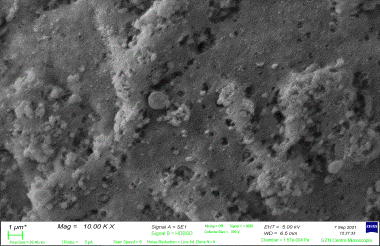

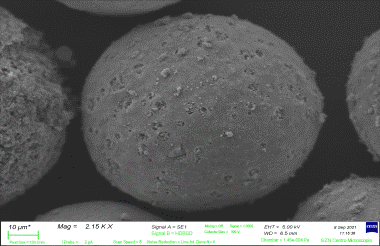

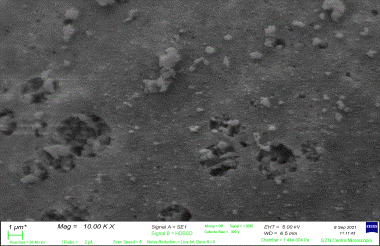

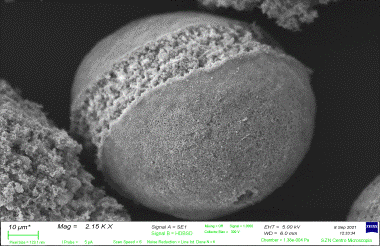

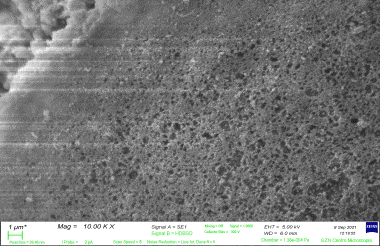


**A**

**B**

**C**

**D**

**E**

**F**

**G**

**H**

**I**

**J**

**K**

**L**

**M**

**N**

Figure 5. SEM images of eggs cryopreserved using droplet vitrification with no CPA (A, B: detail of A); DMSO 0.5M (C, D: detail of C); 1.5M (E, F: detail of E); 3M (G, H: detail of G) or EG 0.5M (I, J: detail of I); 1.5M (K,L: detail of K) and 3M (M, N: detail of M).


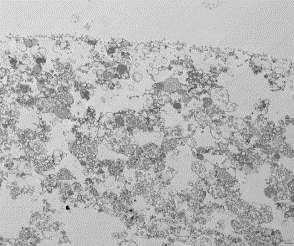

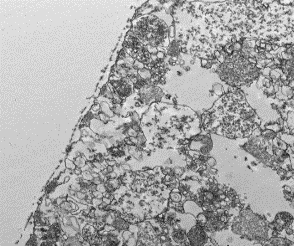

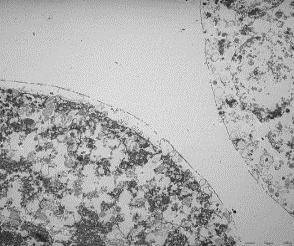

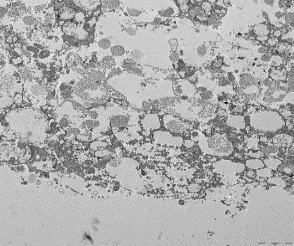

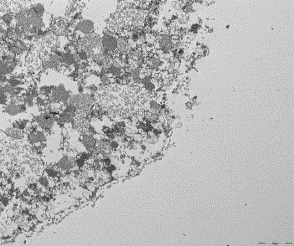

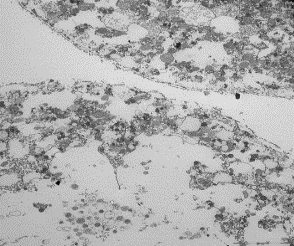

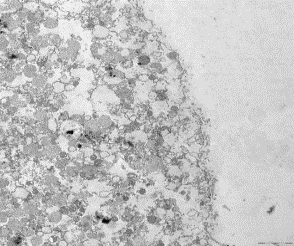


**A**

**D**

**C**

**B**

**E**

**G**

**F**

Figure 6. TEM images of unfertilized eggs cryopreserved using droplet vitrification with no CPA (A); DMSO 0.5M (B); 1.5M (C); 3M (D) or EG 0.5M (E); 1.5M (F) and 3M (G).


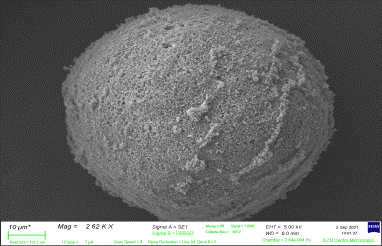

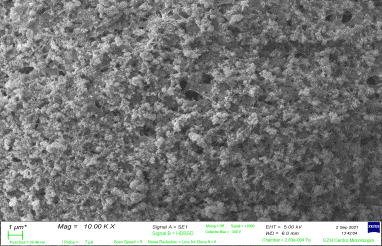

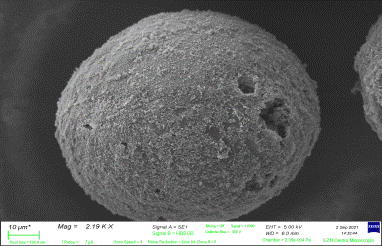

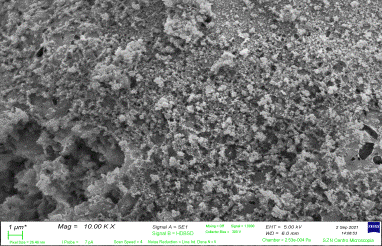

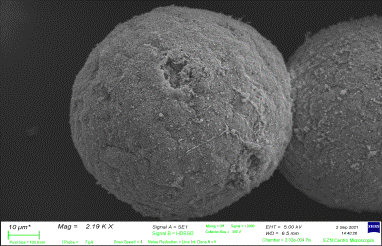

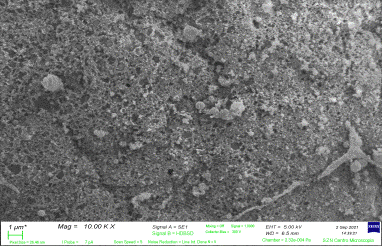

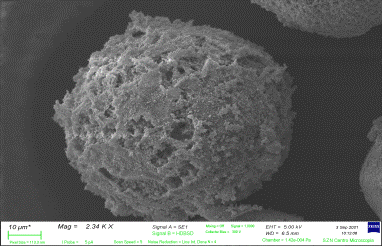

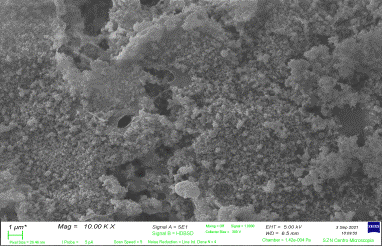

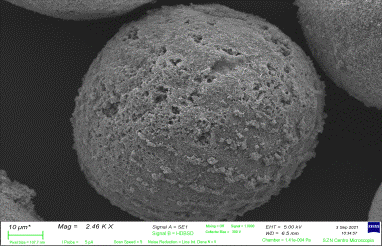

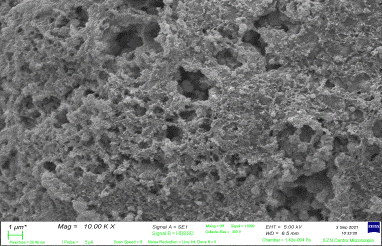

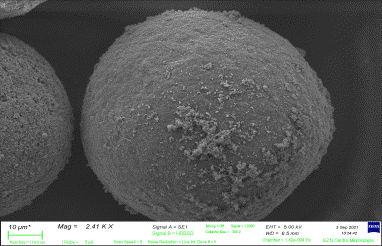

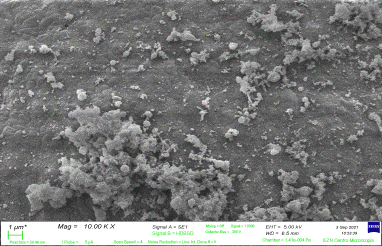

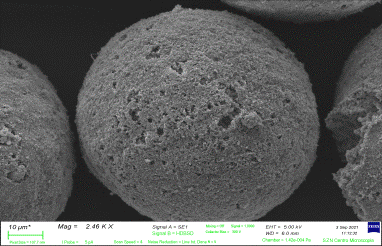

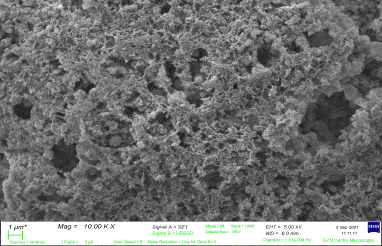


**A**

**D**

**C**

**B**

**I**

**L**

**K**

**J**

**E**

**H**

**G**

**F**

**N**

**M**

Figure 7. SEM images of eggs cryopreserved plunging a straw directly into LN2 with no CPA (A, B: detail of A); DMSO 0.5M (C, D: detail of C); 1.5M (E, F: detail of E); 3M (G, H: detail of G) or EG 0.5M (I, J: detail of I); 1.5M (K, L: detail of K) and 3M (M, N: detail of M).


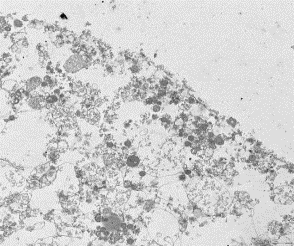

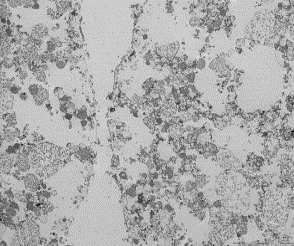

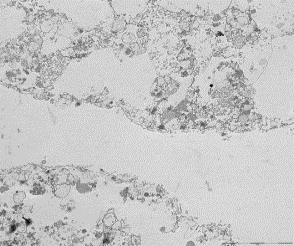

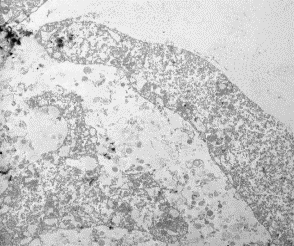

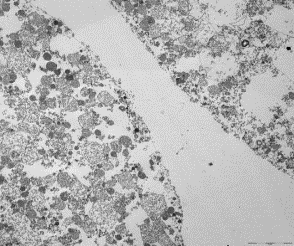

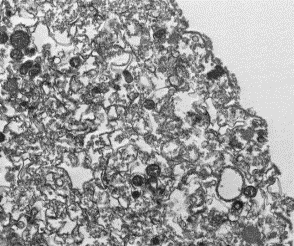

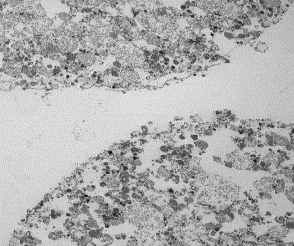


**A**

**D**

**C**

**B**

**E**

**G**

**F**

Figure 8. TEM images of unfertilized eggs cryopreserved plunging a straw directly into LN2 with no CPA (A); DMSO 0.5M (B); 1.5M (C); 3M (D) or EG 0.5M (E); 1.5M (F) and 3M (G).


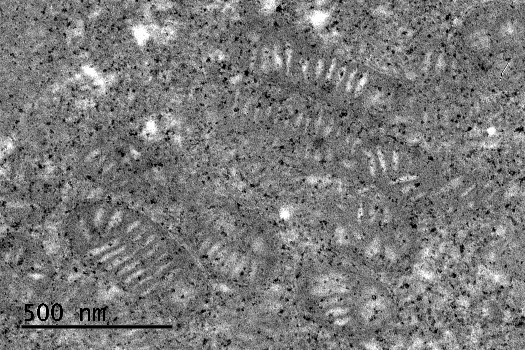

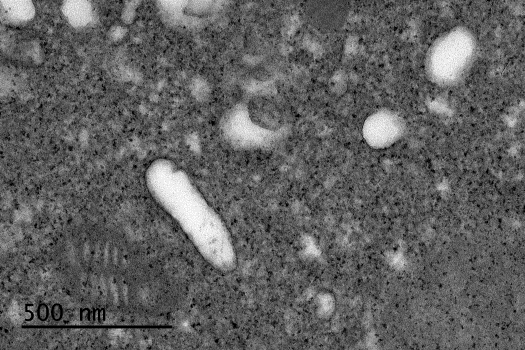


Figure 9. TEM images of detail mitochondria of control eggs.
